# Risk Factors for Severe COVID-19 and Hepatitis C Infections: The Dual Role of Apolipoprotein E4

**DOI:** 10.3389/fimmu.2022.721793

**Published:** 2022-03-11

**Authors:** Felipe B. Lima, Karine C. Bezerra, José Carlos R. Nascimento, Gdayllon C. Meneses, Reinaldo B. Oriá

**Affiliations:** ^1^ Laboratory of Tissue Healing, Ontogeny and Nutrition, Department of Morphology, School of Medicine, Institute of Biomedicine, Federal University of Ceara, Fortaleza, Brazil; ^2^ Division of Anesthesiology, Hospital Geral de Fortaleza, Fortaleza, Brazil; ^3^ Department of Clinical Medicine, Faculty of Medicine, Federal University of Ceara, Fortaleza, Brazil

**Keywords:** COVID-19, liver, apolipoprotein E4, hepatitis C (HCV) infection, inflammation

## Introduction

A new coronavirus, an etiological agent of severe acute respiratory syndrome SARS-CoV-2, was discovered in December 2019 in Wuhan, China, and the coronavirus 2019 (COVID-19) epidemic rapidly escalated globally (1–3). The World Health Organization (WHO) officially declared a pandemic on March 11, 2020; at that time, more than 118 million people got infected worldwide, with the Americas accounting for 44% of the total cases (4).

Currently, one of the most significant issues is to predict who will eventually develop severe illness and even death since this may have implications on public health policies, aiming at preventive actions for specific groups. Risk factors associated with worse outcomes include advanced age, systemic arterial hypertension, diabetes mellitus, ischemic heart disease, obesity, and chronic lung disease (5). However, in many cases, there are no obvious risk factors. Studies are being developed, looking for other associations that could lead to life-threatening outcomes in COVID-19.

Although studies have documented COVID-19 primarily affects the respiratory and endothelial-lining vascular systems, the SARS-CoV-2 may target other organs, such as the liver (6). Different degrees of liver dysfunction are described, mainly inducing transaminases elevation, which is generally transient and mild. No marked increased risk of SARS-CoV-2 infection in patients with chronic liver disease has been observed, although this population is more likely to develop a more severe form of COVID-19, requiring hospitalization, with high morbidity and mortality rates (7, 8).

Dementia seems to contribute as a risk factor for COVID-19 infection outcomes (9–11). This finding raised the hypothesis that other unappreciated risk factors are involved in the pathogenesis of the disease, such as apolipoprotein E (apoE=protein, APOE=gene) since the APOE4, one of the APOE coding-alleles, has a strong association with late-onset Alzheimer’s disease. ApoE is a 299-amino acid protein that binds to plasma lipoproteins and serves as a cholesterol carrier for liver metabolization (12, 13).

Previous research supports that apoE4 protects liver disease progression in hepatitis C virus (HCV)-induced liver injury (14). However, current evidence points to a greater risk for worse COVID-19 in patients carrying this allele, suggesting an ambiguous effect of APOE. To date, no studies have addressed whether chronic liver disease patients carrying the APOE4 gene would have increased risk for more severe COVID-19 infection.

In this opinion paper, we summarize the role of APOE4 on the severity of COVID-19 infection and highlight liver disease outcomes following COVID-19 infection. In addition, we discuss up-to-date findings of APOE4 protection in HCV-induced liver disease.

## COVID-19 Effects on the Liver

First described in early Chinese publications about COVID-19, it was evident that patients with mild disease had alterations in aspartate aminotransferase (AST)/alanine aminotransferase (ALT) 18.2/19.8%, respectively, and severe patients manifested changes of 39.4/28.1% in these transaminases, well-recognized biomarkers of liver dysfunction (2).

Other reviews also highlighted changes in liver enzymes and bilirubin in patients with COVID-19, noting that these manifestations could be multifactorial, including medications, previous liver disease, and even direct viral injury (7, 8), since hepatocytes also have the angiotensin-converting enzyme 2 (ACE2), which the virus binds to enter human cells (15). In addition, the exacerbated inflammatory response triggered by the virus and the hypoxia resulting from ARDS can also contribute as mechanisms of liver damage (16). Monitoring liver function biomarkers is important in all patients diagnosed with COVID-19 to follow the disease evolution.

The association of APOE, HCV infection, and SARS-CoV-2 in liver disease is complex, thus the better understanding of the interrelated injury causal-effects, such as direct viral damage, drug-induced liver injury, hypoxia and microthromboses requires novel clinic and basic research strategies.

SARS-CoV-2 itself can target the liver. Despite the lack of evidence for a specifically targeted mechanism, SARS-CoV-2 may directly or indirectly cause liver damage. The ACE2 receptor, a gateway for SARS-CoV-2 entry in the cells, is highly expressed in cholangiocytes, followed by hepatocytes. Transmembrane serine protease 2 (TMPRSS2), expressed in endothelial cells and involved in SARS-CoV-2 entry and dissemination, is also present in cholangiocytes, erythroid cells, and hepatocytes, sinusoidal endothelial cells of the periportal liver, and less expressed in non-inflammatory macrophages and alpha-beta T cells (17).

The spike protein of SARS-CoV-2 exhibits a unique furin cleavage site, suggesting a role of furin in the pathogenesis of the disease and regulating the efficiency of viral entry. Furin is expressed in hepatocytes and all cell populations present in the liver. Thus, these findings point to the possibility that SARS-CoV-2 can cause liver damage by direct action or by viral cytopathic effect. Also, SARS-CoV-2 can cause liver damage by immune-mediated effects associated with numerous active immune pathways, such as inflammatory macrophages, natural killer cells, plasma cells, mature B cells, and the wide endothelial microenvironment of the liver (17).

Hepatic dysfunction appears to be transient due to mild COVID-19 infection, with satisfactory evolution in most cases, and is rarely associated with permanent liver damage (16). In addition, cirrhosis alone is associated with higher mortality in patients with ARDS (18).

## Novel Findings of Apolipoprotein E4 on COVID-19 Infection

Discovered in the early 1970s, apoE is a glycoprotein expressed in numerous human cells, first described with the crucial function of cholesterol transport and lipid metabolism (19). Located on chromosome 19, the APOE gene is polymorphic in humans. It has three common alleles (E2, E3, E4) responsible for coding different isoforms of this molecule, key for a plethora of biological processes, not only causally linked to lipid transport function, including immunoregulation, tissue repair, and infectious disease-related outcomes (19, 20). Current studies have been documenting the influence of different isoforms of ApoE on viral infections, such as human immunodeficiency virus (HIV), herpes virus (HSV-1), and chronic hepatitis C virus (HCV)-induced liver disease (19, 21, 22).

Since apoE4 is involved in some of other risk factors associated with severe COVID-19, such as atherosclerosis and hypertension (23, 24), there is a growing interest in better understanding how apoE4 immunoinflammatory functions affect the underlying mechanisms associated to severity contributors in SARS-CoV-2 infection. Studies point that APOE4 carriers would show a more intense innate immune response that would lead to more severe systemic inflammation during the Acute Respiratory Distress Syndrome (ARDS) in SARS-CoV-2-infected patients (25). This may partly explain why Afro-descendant Americans are believed to have a more severe disease since they are known to carry the APOE4 allele twice as frequently as European and Asian populations (26). This potential association remains elusive and requires further investigation.

Wang et al. using *in vitro* models identified that apoE4 contributes to the increase in SARS-CoV-2 infection in neuronal and astrocytic cell lineages, suggesting that apoE4-secreting astrocytes play a role in neurological symptoms related to disease severity (27). Other data showed that APOE4 homozygous patients had an independent association with increased risk for severe COVID-19 infection, even when adjusted for preexisting comorbidities, such as dementia, diabetes, and cardiovascular disease (OR> 2.31, 95% CI: 1.65 to 3.24). APOE4 homozygous individuals were 2.2 times more at risk for COVID-19 positivity and 4.3 times more at risk for COVID-19-related lethality than APOE3 homozygous patients (28).

Importantly, apoE is one of the highly expressed proteins in type II alveolar cells in the lungs, where the receptor for SARS-CoV-2 called ACE2 is conspicuous (29). The role of apoE in the lungs is not fully understood and may vary with different pathological conditions. The apoE deficiency in the lung has been related to abnormal lung development in APOE knockout mice. In addition, apoE has both protective and anti-inflammatory properties in the setting of lung disease, reducing primary pulmonary hypertension (30). On the other hand, apoE may lead to pro-inflammatory events in the lung and can function as a concentration-dependent pulmonary danger signal that augments pulmonary inflammatory responses in asthma-related airway conditions (31). In the COVID-19 scenario, yet we do not know whether apoE-related pulmonary danger signals would worsen clinical outcomes in infected patients.

The role of human APOE4 in respiratory infections is poorly explored, especially in COVID-19. The relationship of apoE4 and ACE2 receptors and related-signaling pathways require more investigation. Further studies are needed to investigate the role of APOE4 in pulmonary ACE2 levels and their possible association with worse COVID-19 outcomes after controlling for confounding factors, such as known comorbidities and other ill-related factors.

## Paradoxical Effects of APOE4 on HCV-Induced Liver Disease and COVID-19 Outcomes

Mortality and severity due to COVID-19 are higher in patients with comorbidities, and researchers have documented that the same occurs among those with chronic liver disease. In recent meta-analyses studies, patients with COVID-19 and chronic liver diseases tend to have a more severe SARS-CoV-2 infection [OR 1.48 (95% CI 1.17, 1.87)] and a higher mortality rate [OR 1.78 (95% CI 1.09, 2.93)]. However, chronic liver disease patients are not more often infected with SARS-CoV-2 compared to individuals without this condition (7, 8).

Interestingly, Rhea and colleagues showed that APOE affects radioiodinated S1 (I-S1) uptake in the liver when using APOE target replacement mice. These authors show that male APOE3 mice had the fastest I-S1 uptake in the liver compared with the APOE4 genotype. As the risk of contracting COVID-19 seems greater with APOE4 carriage in humans, these authors suggested that the COVID-19-associated risk seen with APOE4 carriers is unlikely to be due to increased tissue S1 or SARS-CoV-2 uptake (32).

APOE4 allele while predisposing to comorbidities that favor a more severe evolution of COVID-19, such as dementia, hypertension, and ischemic heart disease (33), conversely behaves as a protective factor for some chronic viral-related liver diseases. Studies show that APOE4 patients are more resistant to chronic HCV infection, have a slow progression of liver fibrosis, and are less likely to have alcoholic cirrhosis, non-alcoholic steatohepatitis (NASH) hepatocellular carcinoma (HCC), or virus hepatitis B (HBV) (34–36).

HCV entry into human hepatocytes is a multistep mechanism involving various host factors, including low-density lipoprotein receptor (LDL-R) and heparan sulfate proteoglycans (HSPGs). The lipoviral particle, important for viral infectivity, initially binds to LDL-R and HSPGs through apoE. It has been recognized that the LDL-R is down-regulated in APOE4 carriers (34, 37).

SARS-CoV-2 enters the cell through the binding of the viral spike protein to the ACE2 cell receptor. We speculate that increased apoE4 binding to HSPGs in the lung may enhance SARS-CoV-2 infection, bridging the virus to ACE2 and facilitating viral tissue spread. It has been suggested that HSPGs, such as syndecan, may be an alternative way through which SARS-CoV-2 may enter the lung epithelial cells (38, 39).

ApoE4 is associated with worse cardiovascular outcomes and favors inflammation and obesity that may jeopardize the patient’s health, thus raising the vulnerability to COVID-19 (39, 40). Recent studies provide evidence indicating that apoE4 is associated with coronavirus infection and clinical severity (41).

Knowledge whether carrying apoE4 is more than simply a risk factor, but a pathway for SARS-CoV-2 viral entry and cell infectivity is paramount to identify novel molecular targets for pharmacological intervention.

## Conclusion Remarks

The epidemic of COVID-19 has spread worldwide, and many questions have arisen since then, mainly about the fundamental risk factors involved in the more severe course of the disease. Among the genetic factors studied, the E4 allele of APOE seems to predispose patients to worse outcomes. However, this role is unclear and appears ambiguous when counteracted by some beneficial effects seen in HCV infections and other liver disease conditions. While APOE4 deleteriously affects the pathogenesis of comorbidities that influence the severity of COVID-19, such as dementia, hypertension, and heart disease, paradoxically, APOE4 may be a protective factor against the chronicity of most liver diseases, which could lead to more severe conditions of COVID-19.

Findings of APOE4 deleterious effects on COVID-19 outcomes have been identified in UK biobank studies enrolling patients living in developed settings; however, there is a gap of knowledge whether this potential effect could be replicated in populations living under adverse environments, as APOE4 could have a different role in such conditions (42–44). In addition, APOE4 may be relevant in affecting long COVID-19 cardiovascular sequelae in risk groups (45), which raises public health concerns. Yet we do not know whether HCV-liver injury could increase later cardiovascular effects in APOE4-bearers with long COVID-19.

More studies are needed to dissect the APOE4 immunomodulatory functions related to the deleterious and protective mechanisms seen in different liver viral infections (virus cell entry, viral-induced steatosis and fibrosis, and related-fine inflammatory pathways), which should be better understood to improve disease management and treatment.

## Author Contributions

All authors listed have made a substantial, direct, and intellectual contribution to the work, and approved it for publication.

## Funding

This work was supported by the National Council for Scientific and Technological Development (CNPq), Coordination for the Improvement of Higher Education Personnel (CAPES) grant call CAPES EPIDEMIAS 11/2020, project n° 88881.505364/2020-01, and Fundação Cearense de Apoio ao Desenvolvimento Científico e Tecnológico (FUNCAP) Brazilian funding agencies.

## Conflict of Interest

The authors declare that the research was conducted in the absence of any commercial or financial relationships that could be construed as a potential conflict of interest.

## Publisher’s Note

All claims expressed in this article are solely those of the authors and do not necessarily represent those of their affiliated organizations, or those of the publisher, the editors and the reviewers. Any product that may be evaluated in this article, or claim that may be made by its manufacturer, is not guaranteed or endorsed by the publisher.
